# Derivation of Sendai-Virus-Reprogrammed Human iPSCs-Neuronal Precursors: *In Vitro* and *In Vivo* Post-grafting Safety Characterization

**DOI:** 10.1177/09636897231163232

**Published:** 2023-03-23

**Authors:** Michiko Shigyo, Yoshiomi Kobayashi, Oleksandr Platoshyn, Silvia Marsala, Tomohisa Kato Jr, Naoki Takamura, Kenji Yoshida, Akiyoshi Kishino, Mariana Bravo-Hernandez, Stefan Juhas, Jana Juhasova, Hana Studenovska, Vladimir Proks, Joseph D. Ciacci, Martin Marsala

**Affiliations:** 1 Department of Anesthesiology, School of Medicine, University of California, San Diego, La Jolla, CA, USA; 2 Murayama Medical Center, Department of Orthopaedic Surgery, Tokyo, Japan; 3 Regenerative & Cellular Medicine Kobe Center, Sumitomo Dainippon Pharma Co., Ltd., Kobe, Japan; 4 Division of Stem Cell Medicine, Department of Advanced Medicine, Medical Research Institute, Kanazawa Medical University, Uchinada, Japan; 5 Institute of Animal Physiology and Genetics AS CR, v.v.i., Liběchov, Czech Republic; 6 Department of Biomaterials and Bioanalogous System, Institute of Macromolecular Chemistry, Czech Academy of Sciences, Prague, Czech Republic; 7 Department of Neurosurgery, School of Medicine, University of California, San Diego, La Jolla, CA, USA

**Keywords:** human-induced pluripotent stem cells (hiPSCs), neural precursor cells (NPCs), manual selection, spinal cord grafting, brain grafting, immunodeficient rat

## Abstract

The critical requirements in developing clinical-grade human-induced pluripotent stem cells–derived neural precursors (hiPSCs-NPCs) are defined by expandability, genetic stability, predictable *in vivo* post-grafting differentiation, and acceptable safety profile. Here, we report on the use of manual-selection protocol for generating expandable and stable human NPCs from induced pluripotent stem cells. The hiPSCs were generated by the reprogramming of peripheral blood mononuclear cells with Sendai-virus (SeV) vector encoding Yamanaka factors. After induction of neural rosettes, morphologically defined NPC colonies were manually harvested, re-plated, and expanded for up to 20 passages. Established NPCs showed normal karyotype, expression of typical NPCs markers at the proliferative stage, and ability to generate functional, calcium oscillating GABAergic or glutamatergic neurons after *in vitro* differentiation. Grafted NPCs into the striatum or spinal cord of immunodeficient rats showed progressive maturation and expression of early and late human-specific neuronal and glial markers at 2 or 6 months post-grafting. No tumor formation was seen in NPCs-grafted brain or spinal cord samples. These data demonstrate the effective use of *in vitro* manual-selection protocol to generate safe and expandable NPCs from hiPSCs cells. This protocol has the potential to be used to generate GMP (Good Manufacturing Practice)-grade NPCs from hiPSCs for future clinical use.

## Introduction

The discovery of human somatic cell reprogramming-induced pluripotent stem cells (hiPSCs) technology^1,2^ provided remarkable progress in the potential development of patient-specific cell-replacement-based therapies. Besides, the generation of specific cell types from hiPSCs derived from patients with a defined genetic mutation proved to be a valuable platform for drug target discovery or creating human disease modeling both *in vitro* and *in vivo*^3–6^.

Previous experimental and ongoing clinical studies have established a therapeutic potential of human fetal tissue–derived, embryonic stem cells (ESs)–derived, or iPSC-derived neural precursor cells (hiPSC-NPCs) in the treatment of a variety of neurodegenerative disorders, including spinal cord/brain traumatic injury^7,8^, stroke^9–11^, Alzheimer’s and Parkinson’s disease^12–14^, and ALS^15^. The underlying rationale for using cell replacement therapies is to (1) repopulate the areas affected by neuronal or glial degeneration, (2) provide mechanical support of previously injured tissue, or (3) provide regional neuroprotection by releasing a variety of neurotrophic factors^16–20^.

Several factors are being considered in the process of hiPSC generation particularly if generated cell lines or their lineage-committed derivatives are planned to be used in prospective human clinical trials. First is the source of somatic cells to be used in reprogramming to generate hiPSCs. The most frequently used cell types are skin fibroblasts harvested by biopsies^21^. The key, but relative, limitation in using fibroblasts for reprogramming is the invasive nature to obtain the biopsy material and the need for the initial *in vitro* expansion of collected fibroblasts before the reprogramming process can be initiated^22^. The use of peripheral blood mononuclear cells (PBMCs) has been recently used for reprogramming^23,24^. The primary advantage in using PBMCs is a relative simplicity in obtaining the blood samples and the ability to collect a sufficient number of cells from a single blood draw needed for reprogramming (ie, no need for cell expansion). Also, the collection can be repeated if additional samples are needed. Second, is the use of reprogramming vector(s). The majority of initial studies have employed integrating viral vectors, including retrovirus for reprogramming^1,25^. The major limitation in using integrating vectors for reprogramming is the risk of mutagenesis and resulting tumorigenicity after *in vivo* grafting^26–28^. More recently, the use of several non-integrating vectors to generate hiPSCs from adult somatic cells was reported^29^. One of the non-integrating reprogramming vectors, the Sendai-virus (SeV; a single-stranded RNA virus), has been demonstrated to reprogram somatic cells with high efficiency, low cytotoxicity, and minimal risk of being integrated into the host genome^30^. Using SeV (encoding Yamanaka factors: SOX2, c-MYC, Klf4, and OCT3/4), several research groups generated the hiPSCs by reprogramming human PBMCs^23,24,31,32^. SeV-reprogrammed hiPSCs were further successfully used for the generation of lineage-committed cell line derivatives, including photoreceptor-like cells^33^, and cardiomyocytes^34^.

Here, we report on a successful generation of *in vitro* expandable SeV-hiPSCs-NPCs. Established NPCs show a long-term *in vitro* expandability while maintaining a stable karyotype, expression of neural precursor markers but with a lack of pluripotent markers. Upon transplantation into the striatum or spinal cord of immunodeficient rat grafted NPCs acquire a neuronal and glial phenotype with no detectable tumor formation for up to 6 months post-grafting. These data show that the NPCs manual-selection derivation protocol by using SeV-generated hiPSCs can successfully be used and considered for the future generation of clinical GMP-grade NPCs to be used in prospective clinical trials in the treatment of several neurodegenerative disorders, including spinal ischemic or traumatic injury, ALS, stroke, or brain trauma.

## Materials and Methods

### Development of hiPSC-NPCs

This study was approved by the University of California, San Diego (UCSD) Internal Review Board (IRB) (approval ID 101323).

#### Human-induced pluripotent stem cells

Human peripheral mononuclear cells were isolated from blood samples (33-year-old woman; Lonza, Basel, Switzerland) and reprogrammed by SeV-encoding Yamanaka factors^1,25^. Reprogramming was performed under feeder-free (FF)/xeno-free (XF) condition^35^.

#### Human iPSCs expansion

After reprogramming, cells were plated on Laminin (Laminin 511 E8, iMatrix-511; Nippi, Incorporated. Tokyo; Product code: 892001/892002)-coated six-well plate (cat. no. 3471, CORNING, Corning, NY, USA) in 1.4 × 10^4^ cells/well in iPSC media (AK03N, Ajinomoto, Chuo City, Tokyo, Japan) containing 10 µM ROCK inhibitor (Y37632, Stem Cell Technologies, Cambridge, MA, USA). After 7 days, the hiPSC colonies were dissociated with trypsin solution (2 ml of TripLE solution, Life technologies, Carlsbad, CA, USA; 2 ml of DPBS (Dulbecco’s phosphate-buffered saline); 2 µl of 0.5M EDTA (Ethylenediaminetetraacetic acid)) and passaged. Cells were passaged at least three times before the embryoid bodies (EBs) formation was initiated. A subpopulation of established hiPSCs colonies was used to prepare a single-cell suspension and cells stored in LN vapor for potential future use (AMSBIO, Cambridge, MA, USA).

#### Quantitative polymerase chain reaction assessment of residual SeV

RNAs were prepared from (1) non-infected PBMCs (negative control), (2) SeV-infected PBMCs (3 days post-SeV infection; positive control), and (3) established iPSC line (LPF11; passage 5). cDNAs were synthesized using 1,000 ng of RNAs with Qiagen Quantitect RT Kit (in 20 μl of reaction volume). Quantitative polymerase chain reaction (Qpcr) was performed with TaqMan probes against SeV (custom-made probe targeting the SeV genome located between the coding region of NP and P protein) and β-actin. cDNAs were synthesized from 6.25, 6.25, and 1.56 ng of RNA prepared from negative-control PBMCs, positive-control PBMCs (3 days post-SeV infection), and established iPSC line (LPF11; passage 5), respectively. cDNA of positive control was diluted 160-fold. Conditions of PCR reaction are as follows: 50°C for 2 min, 95°C for 10 min and 40 cycles of (95°C for 15 s and 60°C for 1 min).

#### EB generation

hiPSC colonies were dissociated with trypsin solution (0.05%) and transferred into low attachment six-well plate (CORNING) containing neural induction medium (Stem cell technologies, Cambridge, MA, USA).

#### Rosettes formation

Four to six days *in vitro*–cultured EBs were transferred to poly-L-ornithine/laminin (PLO/L)-coated dishes. Cells were cultured in DMEM/F12-based NPCs media containing 0.5% N2 supplement (Life Technologies), 1% B27 supplement (Life Technologies), 1% glutamate (Life Technologies), and 1% penicillin-streptomycin (Life Technologies) supplemented with 10 ng/ml recombinant human basic fibroblast growth factor (bFGF) (Thermo Fisher Scientific, Waltham, MA, USA) as a solo mitogen.

#### NPCs isolation

Once rosettes-like structures were observed (typically 3–4 days after EBs plating), approximately 100 to 200 morphologically defined NPCs that appeared outside of each rosette were manually picked up by pipette tips and transferred in PLO/L-coating 48-well plates. Isolated NPCs were cultured in the same media as described above for the whole duration of cell expansion. Cells were repeatedly passaged over 10 times. To examine the genetic stability of generated NPCs, karyotype was examined at passage 12 (Cell Line Genetics, Madison, WI, USA).

#### Doubling time

The NPCs were cultured to passage 20, seeded at 2 × 10^5^ cells per well in PLO/L-coated six-well plates. Three wells per day were then separately dissociated with Trypsin and collected in separate tubes to be counted using the Countess II automated cell counter (Thermo Fisher Scientific). The doubling time was calculated using the following equation:

$$\begin{array}{l} DT=t/3.3\times log\left(b/B\right),\\ \mathrm{where}\;DT:\;doubling\;time,\;t:\;time\;in\;hours,\\ b:number\;of\;cells\;at\;the\;end\;of\;the\;point,\\ B:\;the\;number\;of\;cells\;at\;the\;first\;time\;point. \end{array}$$

### Differentiation From the Developed NPCs to Neuron and Glial Cells *In Vitro*

#### NPC differentiation

The NPCs were plated onto chamber slides or coverslips and allowed to reach confluence. The differentiation was initiated with differentiation-induction media (DMEM/F12) containing 0.5% N2 supplement (Life Technologies), 1% B27 supplement (Life Technologies), 1% glutamate (Life Technologies), and 1% penicillin-streptomycin (Life Technologies) supplemented with 10 µg/ml BDNF (brain-derived neurotrophic factor) (PeproTech, Rocky Hill, NJ, USA), 10 µg/ml GDNF (*glial cell line-derived neurotrophic factor*) (PeproTech) with/without 1% fetal bovine serum (FBS). The medium was changed every other day until the cells were fixed with 4% paraformaldehyde (PFA) or used for calcium oscillation assay (~1–4 months after induction).

### Calcium Oscillation in hiPSC-NPCs Derived Differentiated Neurons *In Vitro*

Time-lapse calcium oscillation was analyzed to detect functional neurons in induced hiPSC-NPCs. Four month–induced NPCs cultured on glass coverslips were loaded with Fluo-4AM (reconstituted in DMSO) in Hank’s Balanced Salt Solution (HBSS) for 40 min at room temperature in accordance with manufacturer’s instructions (Invitrogen, Carlsbad, CA, USA). Cells were rinsed with HBSS two times after Fluo-4AM loading and were then incubated at room temperature in the dark for 30 min until data acquisition. Fluorescence imaging of intracellular Ca2^+^ dynamics for periods of 5 min 17 s was performed with a Olympus BX51W1 fixed-stage upright fluorescence microscope (Olympus Corp., Center Valley, PA, USA) equipped with a SPOT-Xplorer™—XS 1.4 MP monochrome camera (Diagnostic Instruments, Inc., Sterling Heights, MI, USA). The imaging system was controlled by SPOT 5.2 ADVANCED Software (Diagnostic Instruments, Inc.). Neurons were imaged in time lapse with a water immersion 40× magnification, 0.8 numerical aperture (NA) LUMPlanFL N objective (Olympus). Images were captured every 1.035 s under low-light level conditions. Images were collected at a slow transfer rate, which reduces background noise, and binned (2 × 2). Digital image processing was performed by using SPOT 5.2 ADVANCED Software (Diagnostic Instruments, Inc.), image processing program ImageJ (NIH, Bethesda, MD, USA) and graphics and data analysis software SigmaPlot 12.5 (Systat Software, Inc., San Jose, CA, USA).

### Animal Studies

All animal studies were approved by the UCSD Institutional Animal care and Use Committee (Protocol No. S01193).

### NPCs Grafting and Differentiation *In Vivo*

#### Intra-striatal cell transplantation

Adult immunodeficient rats (Crl:NIH-Foxn1^rnu^; Charles River; *n* = 8) (Charles River, Wilmington, MA, USA) were anesthetized with isoflurane (5% induction, 1.5%–2% maintenance) and placed in a stereotaxic apparatus to keep the head in a fixed position. After the scalp was shaved, a sagittal midline skin incision was performed to expose the skull. To permit an intra-parenchymal brain injection, a small borrow hole was drilled into the skull using a dental drill. The NPCs (30,000 cells/µl/injection) were injected into the striatum (stereotaxic X-Y-Z coordinates: bregma 0.5 mm, lateral to ± 3.0 mm, and 3.8, 5.0, and 6.2 mm depth: three injections delivered at each depth bilaterally) using a 34G needle interconnected with a digital microinjector (Tritech Research, San Diego, CA, USA).

#### Spinal cord parenchyma cell transplantation

After laminectomy at the T13-L1 level, rats (Crl: NIH-Foxn1^rnu^; Charles River; *n* = 8) received five bilateral spinal cord gray matter injections of NPCs (30,000 cells/µl/injection; X-Y-Z coordinates: lateral to midline ± 0.8 mm and ~ 1.0 mm depth). Cells were injected by using a Hamilton Syringe and homemade glass capillary (inner diameter: 80 µm, outer diameter: 100 µm) interconnected with a digital microinjector (Tritech Research).

Same animals received both intra-spinal and intra-cerebral NPCs injections.

### Animal Perfusion-Fixation and Immunofluorescence Staining

At 2 months (*n* = 3) or 6 months (*n* = 5) after *in vivo* NPCs grafting rats were terminally anesthetized with pentobarbital (100 mg/kg; i.p.) and transcardially perfused with heparinized saline (100 ml) followed by 4% PFA in PBS. The brain and spinal cord tissues were then dissected, post-fixed with 4% PFA overnight at 4°C, and cryoprotected in 30% sucrose for a minimum of 5 days. Coronal brain or transverse spinal cord sections were then cut on a cryostat (20–30 µm thick) and stained by using a standard immunofluorescence (IF) protocol. Sections were first washed with 0.3 % Triton-100-containing PBS (TX-PBS) 3 × 10 min and incubated with a blocking solution containing 5% normal donkey serum in 0.3% TX-PBS for 60 min. This was followed by incubation in primary antibodies (Table 1) in blocking solution overnight at 4°C. Sections were then washed 3 × 10 min in PBS and incubated with fluorophore-conjugated secondary antibodies diluted in 0.3% TX-PBS for 1 h. After 3× wash with PBS, nuclear staining was performed using DAPI solution (0.1 µg/ml) followed by three times rinses in PBS. Sections were then mounted on slides and covered with anti-fade mounting medium (ProLong; Thermo Fisher Scientific).

**Table 1.** Antibodies Used for Flow Cytometry and Immunofluorescence Staining.

Catalog no.	Name	Company
Antibodies used for flow cytometry		
560341	Nestin—Alexa Fluor 647	BD Biosciences
555427	CD24—FITC	BD Biosciences
561664	PAX6—Alexa Fluor 488	BD Biosciences
561610	Sox2—V450	BD Biosciences
560791	Nanog—Alexa Fluor 488	BD Biosciences
Primary antibodies used for immunofluorescence staining		
AF1759	OCT-3/4	R&D systems
MAB1435	SSEA4	R&D systems
AB5603	SOX2	Millipore Sigma
AF1997	Nanog	R&D systems
MAB6326	Nestin	Millipore Sigma
610920	N-cadherin	BD Biosciences
339100	ZO1	Invitrogen
ab104854	Plzf	abcam
AB5934	SOX1	Millipore Sigma
AF1979	Otx2	R&D Systems
AF8150	Pax6	R&D Systems
134014	NeuN	Abcam
MAB377	NeuN	Millipore Sigma
ABN78	NeuN	Millipore Sigma
S4403	MAP2	Sigma
Sc-8066	DCX	Santa Cruz
G3893	GFAP	Sigma
AB5541	GFAP	Chemicon
TA302094	Human GFAP	Origene
50-6525-80	Synaptophysin	eBioSciences
VP-S285	Synaptophysin	Vector laboratories
Sc-73650	GAD65	Santa Cruz biotechnology
AB5905	VGLUT1	Millipore Sigma
AB2251-1	VGLUT2	Millipore Sigma
AB5421-1	VGLUT3	Millipore Sigma
Ab9610	Olig2	Millipore Sigma
AB5733	Vimentin	EMD Millipore
Ab16667	Ki67	abcam
Ab36999	Human NuMA	abcam
AB951	Human NSE	Chemicon
A-2052	GABA	Sigma
147011	Gephyrin	Synaptic Systems
ab13970	GFP	abcam
AB144P	ChAT	Millipore Sigma
131002	VGAT	Synaptic Systems

For *in vitro* cultured cells, NPCs plated on glass coverslips were fixed in 4% PFA for 20 min and washed 3 × 5 min in PBS. After blocking with 5% normal donkey serum for 30 min, cells were incubated with primary antibodies (Table 1) in blocking solution overnight at 4°C. After 3 × 5 min rinses in PBS, the cells were then incubated with fluorophore-conjugated secondary antibodies in 0.2% TX-PBS for 1 h followed by 3 × 5 min rinses in PBS. Nuclei were stained with DAPI solution (0.1µg/ml) for 3 min followed by 3 × 5min wash in PBS. Coverslips were then mounted on slides with anti-fade mounting medium (ProLong; Thermo Fisher Scientific). The images were acquired using fluorescence (Zeiss Axio Imager M2 Microscope(Zeiss, Oberkochen, Germany) with Stereo Investigator software (MBF Bioscience, Williston, VT, USA), and using a confocal microscope (Fluoview FV1000, Olympus) with Olympus FV10-ASW Viewer software.

### Flow Cytometry

Flow cytometry analysis was performed by following the company’s instruction with modification (BD Biosciences, San Jose, CA, USA). At first, the established NPCs were cultured in DMEM/F12 media supplemented with 10 ng/ml bFGF as described before. Once cells reached confluence, cells were dissociated with Accutase (cat. no. AT-104, Innovative Cell Technologies co., San Diego, CA, USA) and a single-cell suspension (1 × 10^6^ cells per 1 ml sample) prepared and incubated with Aqua dye (no. L34965, Thermo Fisher Scientific) to detect live/dead cells for 30 min. After washing the cells with FACS buffer consisting of 97% DPBS, 2% FBS, and 1% (5 mM) EDTA, cells were fixed with 4% PFA in PBS. Then, permeabilization was done with a buffer (BP Phosflow Perm. Buffer III, cat. no. 558050, BD Biosciences) for 15 min. Cells were washed with FACS (*fluorescence-activated cell sorting*) buffer 2 ×, blocked with 5% normal donkey serum in FACS buffer, and then incubated with each antibody (Table 1) for 30 min in the dark. Expression of extracellular and intracellular protein markers was detected using flow cytometry using BD LSRFortessa (BD Biosciences). The data were analyzed by Flowjo analysis software (FLOWJO, Ashland, OR, USA).

### Statistical Analysis

All data are reported as the mean ± SEM. An unpaired two-tailed Student’s *t* test was used for single comparisons of grafted cells at the 2-month and 6-month post-grafting. In each case, *P < 0.05 and **P < 0.01 were considered to be statistically significant. The GraphPad Prism software (version 6.0c; GraphPad Software Inc., San Diego, CA, USA) was used for all analyses.

## Results

### Lack of Residual SeV in Established Pluripotent LPF11 Cells

Using qPCR, we first probe for the residual presence of SeV (targeting the SeV genome located between the coding region of NP and P protein) in established pluripotent LPF11 cells. Compared with positive control (PBMCs 3 days post-SeV infection), no residual SeV was detected (Supplementary Fig. 1).

### Generation of NPCs from hiPSCs

**Figure 1.**
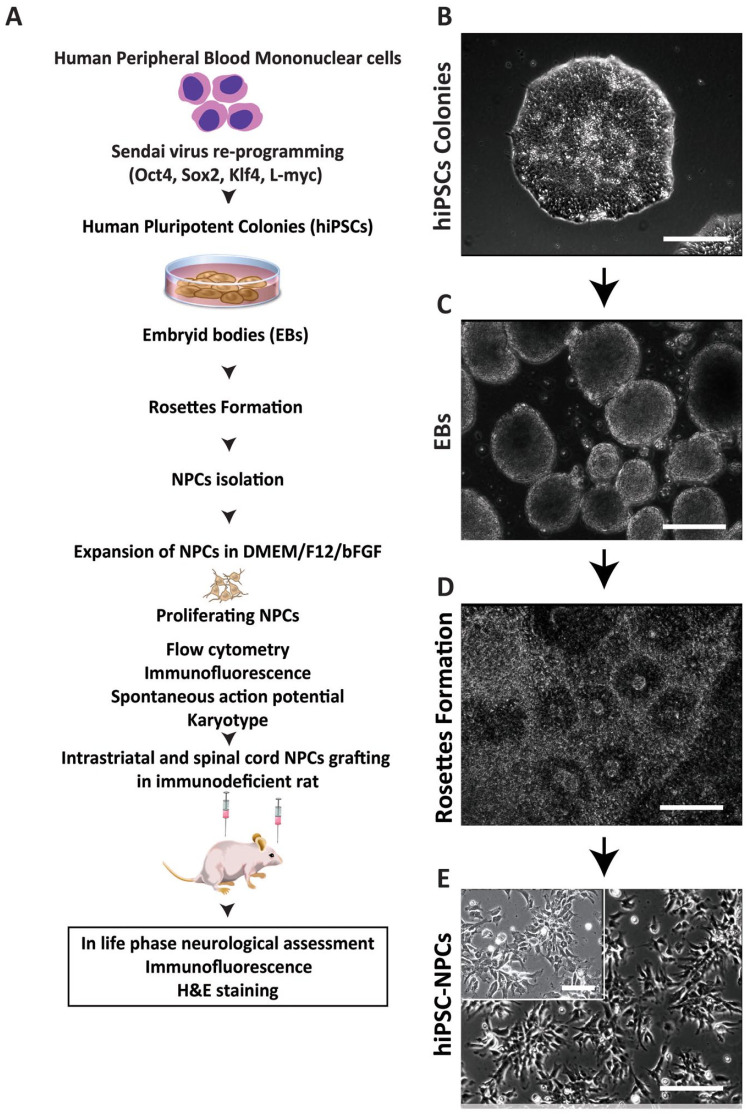
Strategy for development and *in vitro* and *in vivo* characterization of hiPSC-NPCs. (A) Schematic diagram depicting the experimental design of *in vitro* hiPSC-NPCs generation and *in vitro* and *in vivo* cell characterization. (B) Morphology of established hiPSCs colony. (C) Morphology of free-floating EBs. (D) A colony of neural rosettes cultured on P/L coated dish. (E) Representative image of established hiPSCs-NPCs. Scale bars: 100 µm (B, C, D, E). hiPSCs-NPC: human-induced pluripotent stem cells–derived neural precursors; EB: embryonic body, DMEM (Dulbecco’s Modified Eagle Medium).

To generate the hiPSC-NPCs, a previously developed protocol established to isolate the NPCs from human pluripotent ESs and porcine iPSCs was employed^36,37^. First, the peripheral mononuclear blood cells were isolated from human blood samples and reprogrammed by SeV vectors encoding octamer-binding transcription factor 4 (OCT4), (sex-determining region Y)-box2 (SOX2), Kruppel-like factor 4 (KLF4), and myelocytomatosis viral oncogene (L-MYC). After reprogramming, cells were cultured on matrix-coating dishes as a monolayer (Fig. 1A, B).

Established pluripotent colonies (Fig. 1B) were manually dissociated and transferred into low attachment dishes to induced EBs (Fig. 1C). After 4 to 6 days, established EBs were transferred onto PLO/L pre-coated dishes and induced using NPCs induction media (see “Material and Methods” for details). Attached EBs gradually flattened in shape and the appearance of neural rosettes (NRs) was seen in about 4 to 7 days after EBs plating/induction (Fig. 1D).

Several cell clusters originating from NRs but localized at the edges of NRs were then manually picked and re-plated to P/L coating dishes. These cell populations were passaged several times until morphologically defined NPCs-like cell groups were identified. Colonies of NPCs-like cells were then manually harvested, expanded on P/L coated dishes (Fig. 1E), and used in all subsequent *in vitro* and *in vivo* grafting experiments (Fig. 1A).

**Figure 2.**
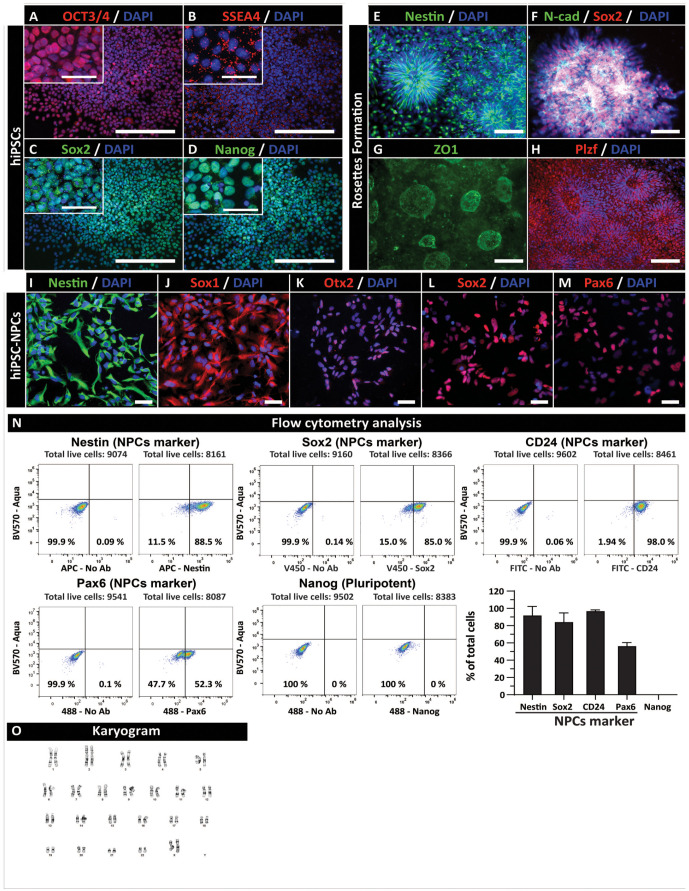
*In vitro* characterization of pluripotent and hiPSC-derived NPCs. Immunofluorescent staining of pluripotent hiPSCs colonies with (A) OCT4, (B) SSEA4, (C) SOX2, and (D) Nanog. Established neural rosette(s) expressing (E) Nestin, (F) (N-cadherin; N-cad), Sox2 (G) ZO1, and (H) Plzf. Proliferating NPCs at passage 19 stained with (I) Nestin, (J) Sox1, (K) Otx2, (L) Sox2, and (M) PAX6 antibodies. (N) Flow cytometry analysis of proliferating NPCs at passage 20 shows the consistent presence of NPC markers (Pax6, SOX2, CD24, and Nestin) but lack of pluripotent marker Nanog. (O) Normal karyogram in established NPCs (passage 12). Scale bars: 50 µm (A–D) and 20 µm (E–M, A–D-inserts). hiPSC: human-induced pluripotent stem cell; NPC: neural precursors.

The expression of pluripotent markers, NRs-associated markers, and proliferating NPCs markers was analyzed periodically during the process of NPCs generation by IF and flow cytometry. In established pluripotent colonies, iPSC marker(s) expression, including OCT3/4, SSEA4, SOX2, and Nanog, was consistently seen (Fig. 2A–D). Staining of NRs showed a high level of expression for Nestin, N-cadherin, Sox2, ZO1, and Plzf (Fig. 2E–H). Tight junction protein ZO1 was detected asymmetrically in the central part of rosettes formations (Fig. 2G). Analysis of NPCs markers, including nestin, SOX1, OTX2, SOX2, and PAX6 showed consistent presence of all markers in established (passage 19) NPCs (Fig. 2I–M). Quantitative flow cytometry analysis of NPC markers showed 52.20% Pax6, 84.86% SOX2, 97.94% CD24, and 88.41% nestin-positive cells. No expression of the pluripotency marker (Nanog) was seen (Fig. 2N). Established NPCs (passage 12) showed normal karyotype (Fig. 2O).

### Established hiPSCs-NPCs Differentiate Into Functional Neurons and Glial Cells *In Vitro*

A defining characteristic of multipotent NPCs is their ability to generate functional neurons and glial cells (astrocytes and oligodendrocytes) after differentiation. To probe for the multi-lineage potency of established NPCs (passages 11–15), cells were treated with differentiation media (containing 10 ng/ml BDNF and 10 ng/ml GDNF) for 2 months. Induced cells were analyzed by (1) IF using neuronal and glial markers, (2) *in situ* fluorescence hybridization to identify glutamatergic or GABAergic mRNA transcripts, and, (3) by calcium oscillation imaging to confirm the presence of action potential-generating neurons.

**Figure 3.**
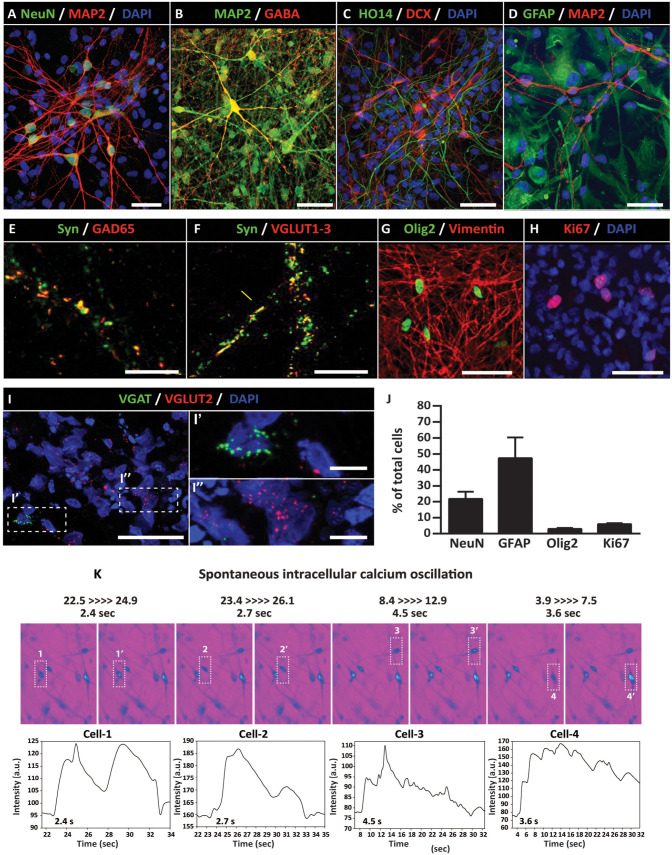
Induced hiPSC-NPCs generate functional neurons *in vitro*. Immunofluorescence staining of 2 month–induced NPCs show expression of neuronal markers including (A) NeuN/MAP2, (B) MAP2/GABA, and, (C) HO14/DCX. (D) Expression of astrocyte marker (GFAP). (E, F) Co-localization of Syn/GAD65 and Syn/VGLUT1-3 in synaptic terminals. (G) Expression of Olig2 and Vimentin in immature glial cells. (H) Ki67 (mitotic marker) is seen in a small subpopulation of cells. (I) FISH shows the presence of VGLUT2 and VGAT transcripts in a separate population of induced neurons. (J) Quantification of NeuN, GFAP, Olig2, and mitotically active Ki67+ cells. (K) Presence of spontaneous intracellular calcium oscillation in induced neurons at 4 months after differentiation. Scale bars: 50 µm (I), 10 µm (A, B, C, D, E, F, I′, I″), 5 µm (G, H). DCX: doublecortin; GFAP: glial fibrillary acidic protein; hiPSCs-NPC: human-induced pluripotent stem cells–derived neural precursors; NeuN: DNA-binding neuron-specific protein; VGAT: vesicular GABA transporter.

At 2 months after differentiation in neuron-inducing media, a subpopulation of cells formed radiated neuron-like morphology and showed expression of immature- (DCX—doublecortin) and mature- (MAP2—microtubule-associated protein 2), (NeuN—DNA-binding neuron-specific protein) and (HO14-human-specific axonal neurofilament) neuronal markers (Fig. 3A–D). Staining with neurotransmitter phenotypic markers shows co-localization of synaptophysin+ puncta with GAD65 (GABAergic marker) and VGLUT1-3 (glutamatergic marker) (Fig. 3E, F). Staining with astrocyte marker [glial fibrillary acidic protein (GFAP)], oligodendrocyte marker (Olig2), and early glial marker (vimentin) shows a consistent presence of all three markers (Fig. 3D, G). Ki67 (mitotic marker) immunoreactivity was seen in a small population of cells scattered through the analyzed cell population (Fig. 3H). Staining with GABA antibodies showed well-established GABAergic neurons (Fig. 3B). FISH analysis using VGLUT2 (glutamate transporter 2) and VGAT (vesicular GABA transporter) probes confirmed the presence of both excitatory glutamatergic and GABAergic neurons (Fig. 3I). Quantitative analysis of relative numbers of NeuN+, GFAP+, OLIG2+, and Ki67+ cells is presented in Fig. 3J.

To study the ability of induced neurons to generate action, potential cells were loaded with Fluo-4 AM and spontaneous calcium oscillation measured using high-resolution fluorescence microscopy. Numerous neurons with calcium oscillation patterns consistent with spiking neurons (ie, periodic, quasi-periodic, or chaotic spiking’s, as well as bursting’s activities) were seen (Fig. 3K).

Taken together, these data demonstrate that established NPCs have multi-lineage potential and can generate mature-functional excitatory inhibitory neurons and glial cells (astrocytes and oligodendrocytes).

### *In Vivo* Grafted NPCs Are Non-Tumorogenic and Differentiates Into Neurons and Glial Cells in the Striatum and Spinal Cord of the Immunodeficient Rat

To characterize the safety and differentiation potential of established NPCs, cells were grafted into striata or lumbar spinal cord in the adult immunodeficient rat. Animals were sacrificed at 2 or 6 months post-grafting and the presence of grafted cells analyzed by H&E staining or IF using human-specific and non-specific antibodies (Fig. 1A).

IF analysis of NPCs-grafted striata and spinal cords at 2 months postgrafting showed a consistent presence of grafted human cells stained with a human-specific nuclear marker (hNUMA), (Supplementary Figs. 2A and 3A). Staining with early (DCX) and late (NeuN, hNSE, and HO14) neuronal markers showed the presence of all neuronal markers in individual grafts. In general, the most intense expression was seen for early neuronal marker DCX and relatively lower expression was seen for late neuronal markers (NeuN, hNSE) (Supplementary Figs. 2A–E and 3A–C). Moderate intensity of human-specific synaptophysin staining was seen throughout the grafts with some hSYN+ puncta associated with human axons (HO14) (Supplementary Figs. 2C, D, and 3B). Staining with VGAT (pre-synaptic inhibitory marker) and gephyrin (post-synaptic glycine receptor-associated protein) showed the presence of putative synaptic inhibitory contacts between grafted neurons and neurons of the host (Supplementary Figs. 2F and 3F). Analysis of glial markers showed a relatively low expression of GFAP and Olig2 but with a high level of expression for vimentin (early glial marker) (Supplementary Figs. 2A, B, G, H, and 3A, C, D, E).

**Figure 4.**
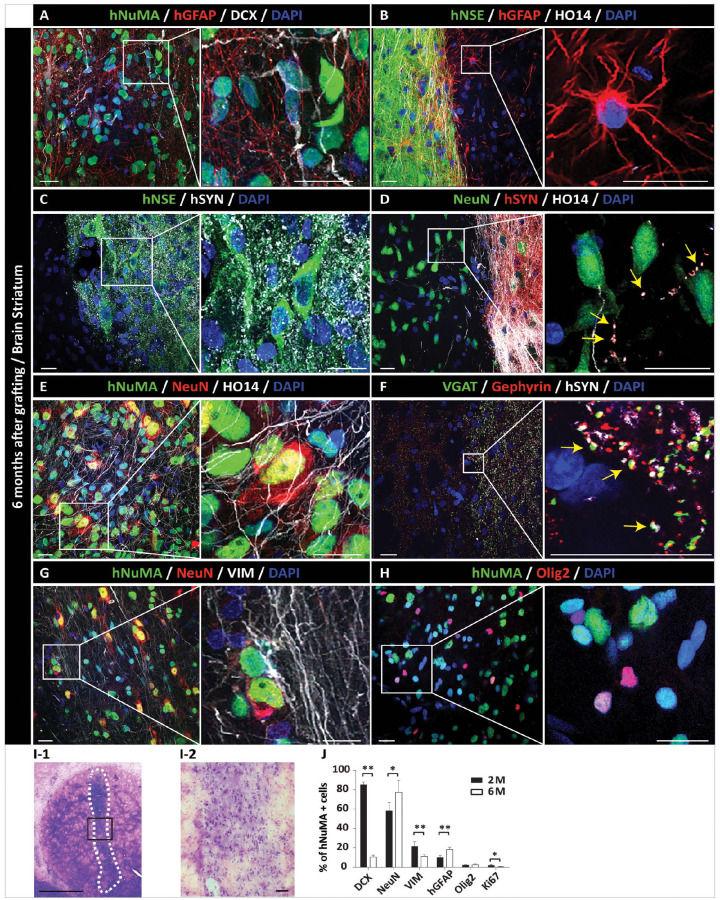
Long-term (6 months) engraftment and neuronal and glial differentiation of hiPSC-NPCs after grafting into striata of the immunodeficient rat. (A) IF analysis of the coronal brain section showed a consistent presence of hNUMA+ grafts in the targeted striatal region. Staining with human-specific GFAP shows the homogenous distribution of grafted NPC-derived astrocytes throughout the graft. (B) Intense human-specific NSE expression (green area) with well morphologically defined human astrocytes (red signal). (C) Areas occupied with mature grafted human neurons (hNSE) show intense human-specific synaptophysin (hSYN) expression. (D) hSYN expression is primarily present in areas with a high density of grafted neuron-derived human axons (HO14+), (yellow arrows). (E) Expression of mature neuronal marker NeuN in hNUMA+ grafted neurons. (F) A high density of VGAT punctate-like expression in areas of hSYN immunoreactivity. hSYN+ terminals show co-localization with VGAT immunoreactivity and are opposed to post-synaptic gephyrin immunoreactivity (yellow arrows). (G) Presence of vimentin+ glial progenitors at the periphery of hNUMA+ graft. (H) Expression of Olig2 (oligodendrocyte marker) immunoreactivity in hNUMA+ grafted cells. (I) Hematoxylin-eosin-stained section with normally appearing graft. No hyper-cellularity (indicative of tumor formation) can be identified (I-1, I-2). (**J**) Quantitative analysis of neuronal and glial marker(s) expression. Scale bars: 20 µm (A–H), 10 µm (A–H; inserts). *P < 0.05; **P < 0.01, all data are presented as mean ± SEM, *n* = 3, two-tailed *t* test. DCX: doublecortin; GFAP: glial fibrillary acidic protein; hiPSCs-NPC: human induced pluripotent stem cells–derived neural precursors; hNUMA: human-specific nuclear marker; IF: immunofluorescence; NeuN: DNA-binding neuron-specific protein; SEM: standard error of the mean; VGAT: vesicular GABA transporter.

**Figure 5.**
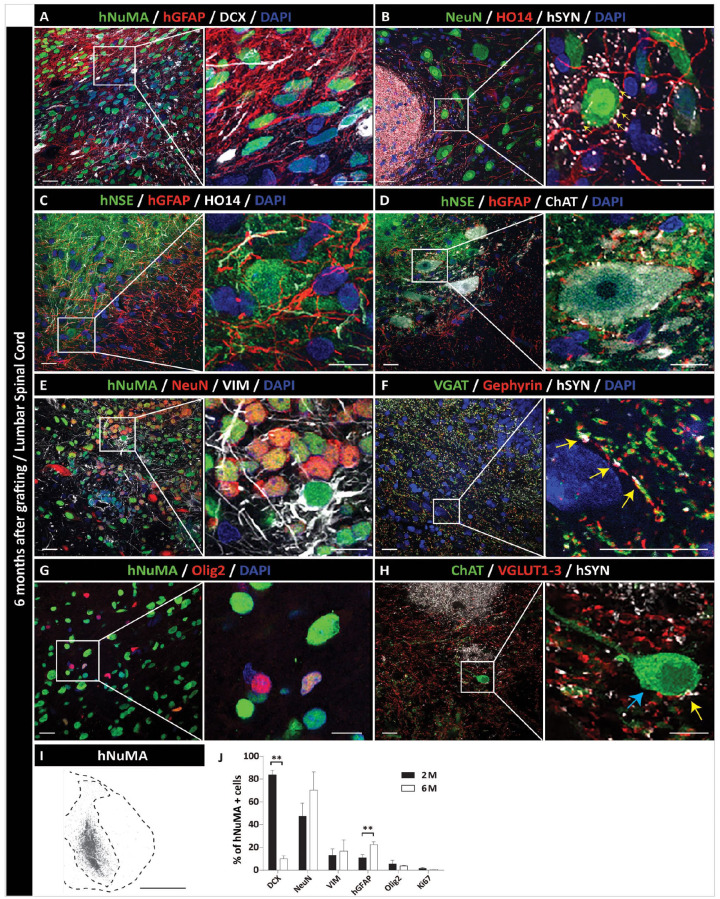
Long-term (6 months) engraftment and neuronal and glial differentiation of hiPSC-NPCs after grafting into the lumbar spinal cord of the immunodeficient rat. (A) IF staining of transverse lumbar spinal cord section with hNUMA antibody shows the homogeneous distribution of grafted cells. In the same areas, a high density of human astrocytes (hGFAP+ cells) and residual DCX immunoreactivity is seen. (B) Intense human-specific synaptophysin (hSYN) immunoreactivity within the graft. A dense axonal network (HO14) derived from grafted cells and expressing hSYN can also be seen. (C, D) Expression of mature neuronal marker (hNSE), glial marker (hGFAP), and human axonal neurofilament (HO14). (E) NeuN expression in hNUMA+ grafted neurons in the core of the graft. Vimentin immunoreactivity in the same region can also be seen. (F) Co-localization of hSYN puncta with VGAT immunoreactivity in the proximity of post-synaptic gephyrin+ puncta (yellow arrows). (G) Expression of Olig2 (mature oligodendrocyte marker) in grafted hNUMA+ cells. (H) Expression of VGLUT1-3 immunoreactivity in grafted neuron-derived hSYN puncta (yellow arrows) and residing on the CHAT+ α-motoneuron of the host (blue arrows). (I) hNUMA positive graft (black color–stained cells), representing a single injection site localized between the center of the dorsal horn and ventral horn. (J) Quantitative analysis of neuronal and glial marker(s) expression. Scale bars: 20 µm (A–H), 10 µm (A–H; inserts). **P < 0.01, all data are presented as mean ± SEM, *n* = 3, two-tailed *t* test. DCX: doublecortin; GFAP: glial fibrillary acidic protein; hiPSCs-NPC: human induced pluripotent stem cells–derived neural precursors; hNUMA: human-specific nuclear marker; IF: immunofluorescence; NeuN: DNA-binding neuron-specific protein; SEM: standard error of the mean; VGAT: vesicular GABA transporter.

IF analysis at 6 months post-grafting showed a more advanced stage of grafted NPCs maturation in both striatum and spinal cord. Staining with mature neuronal markers (hNSE and NeuN) showed a high number of intensely hNSE-stained and NeuN-stained neurons throughout the grafts (Figs. 4B, C, E, G and 5C, D, E). An intense hSYN in the graft and often associated with peripherally projecting HO14+ human axons was also seen (Figs. 4C, D, and 5B). Co-staining with hSYN and VGAT or VGLUT1-3 showed the presence of both inhibitory (VGAT+) and excitatory (VGLUT1-3) puncta co-localizing with grafted neurons-derived hSYN+ terminals (Figs. 4F and 5F, H). Compared with 2 months post-grafting, the density of GFAP+ astrocytes was clearly increased in the graft as well as at the border of the graft with the host tissue (Figs. 4A, B, and 5A, C). In the same regions, a continuing presence of early glial marker vimentin was seen, suggesting an ongoing glial proliferation (Figs. 4G and 5E). Oligodendrocyte staining with Olig2 antibody (co-staining with hNUMA) showed the majority of double-stained grafted cells within the grafts (Figs. 4H and 5G). Hematoxylin and eosin (H&E) staining of striatal sections showed normally appearing mature neural grafts with no sign of hyper-cellularity (which would be indicative of tumor formation) (Fig. 4I-1, 2). Quantitative analysis of DCX-, NeuN-, vimentin-, hGFAP-, Olig2-, and Ki67-positive cells at 2 and 6 months is provided in Figs. 4J and 5J.

## Discussion

Using SeV-reprogrammed hiPSCs, we demonstrate an effective generation of NPCs by using a manual-selection protocol. Established NPCs line can be expanded for over 20 passages while maintaining a stable karyotype, expression of neural precursor markers, and with no signs of spontaneous differentiation. No expression of pluripotent markers was seen in established NPCs. *In vitro*–induced NPCs show differentiation toward glial phenotype (oligodendrocytes and astrocytes) and excitatory and inhibitory neurons capable of generating spontaneous action potentials. Established NPCs grafted into striata or spinal cord of immunodeficient rats show robust engraftment and neuronal and glial differentiation at 2 or 6 months post-grafting. No tumor formation was seen in NPCs-grafted regions.

### Strategies for Purification/Enrichment and Expansion of NPCs From Pluripotent Stem Cells

Several previous studies have demonstrated the successful use of fluorescence-activated cell sorting, by using several combinations of neural and/or neuronal cell surface markers to isolate/enrich the population of expandable neural/neuronal precursors^38–43^. While effective in generating well-defined NPC populations, these protocols require the use of GMP-grade antibodies and cell-sorting equipment(s). As such, routine use of the FACS protocol for clinical use is prohibitively expensive. In our previous study, we have developed and characterized a manual-selection protocol that permits a reliable morphologically defined selection of NPCs from human ES cell lines^37^. Our current study, which employed the same protocol, demonstrates that the NPCs can similarly be isolated from established hiPSCs. Comparably, as previously shown for ES-derived NPCs, a stable karyotype and lack of pluripotent markers were seen after long-term passaging of hiPSCs-NPCs. Mature neurons derived from established NPCs showed action potential-generating properties and expression of both excitatory and inhibitory neurotransmitter phenotype. These data are similar to other studies that show the appearance of functional neurons after induction of porcine iPSCs-NPCs *in vitro*^36^ or long-term maintained hiPSC-derived brain organoids^44,45^.

### Selecting the Optimal *In Vitro* NPCs Culture Protocol to Generate Transplantable NPCs Cell Bank

One of the key determinants defining a successful and effective translation of cell-replacement-based therapies into clinical practice is the format on how the established NPCs cell lines are cultured, stored, and prepared for *in vivo* grafting. In general, two basic protocols are being employed to culture and expand NPCs whether derived from fetal central nervous system (CNS) tissue, ESs, or iPSC lines. First, the NPCs are cultured in the form of neurospheres^46^. For passage, the individual neurospheres are enzymatically or mechanically dissociated and then subcultured. While the *in vitro* culturing of established neurospheres is relatively straightforward and does not require any coating of tissue culture wells, there are three key limitations from the perspective of effective use of neurospheres in a clinical setting. (1) The freezing of established neurospheres is typically associated with a poor post-freezing cell/neurospheres recovery thus limiting the use of previously frozen neurospheres for routine clinical use. (2) Because the size of individual neurospheres varies significantly the required cell dosing to be delivered is difficult to control unless the filtering of prepared neurospheres culture is performed before grafting. (3) Finally, a relatively large size of neurospheres and tendency of aggregation prohibit the use of small diameter needles (~30G) for intra-parenchymal delivery. This can be of particular concern when the spinal delivery of cells (which requires smaller needles relative to brain injection cannulas) is desired^47–49^. Second, the NPCs can be cultured as a cell monolayer on previously coated tissue culture wells. For coating, a combination of PLO/L is typically used^50^. This culturing protocol has successfully been used to expand and bank clinical-grade NPCs derived from fetal tissue or ESs lines^49,51^. Our current protocol used a comparable approach with NPCs expanded as a monolayer on a previously P/L-coated surface. While relatively more laborious, this protocol has several specific advantages (compared with neurosphere technology) once considered for potential clinical application: (1) a consistent neural precursor cell morphology can be seen in established-proliferating NPCs, (2) minimal or no spontaneous differentiation is typically seen even with extensive passaging, (3) over 90% cell viability is usually seen in previously frozen cells after thawing, and (4) a relatively smaller injection needle (~30g) can be used without difficulties to deliver single-cell suspension into the brain or spinal cord in a large animal model(s) (such as pig or NHP) and in human^36,47–49,52,53^. As such, the use of monolayer NPCs culturing and expansion is a preferable method in producing NPC cell bank(s) intended for clinical use.

### Manually Selected and *In Vitro*–Expanded hiPSCs-NPCs Show Long-Term *In Vivo* Engraftment in the Absence of Tumor Formation

In our current study, *in vivo* grafted NPCs into striata and spinal cord of immunodeficient rats showed a robust neural differentiation with the presence of mature neurons and glial cells (oligodendrocytes and astrocytes). No tumor formation was seen at 2 or 6 months post-grafting. Mature grafted neurons showed extensive axodendritic sprouting and development of putative inhibitory and excitatory synaptic contacts with the host interneurons and CHAT+ α-motoneurons. Qualitatively, these data are very similar to the differentiation profile of human fetal spinal cord-, ES-, or iPSCs-derived NPCs grafted spinally in naïve immunosuppressed adult pig or immunodeficient rat^53,54^, spinally injured rat or non-human primates^52,55–58^, or in rat with previous spinal ischemic injury^59,60^. Similarly as shown in previous studies, no excessive proliferation of grafted cells was seen and the only occasional presence of grafted Ki67+ cell was detected. Jointly these data demonstrate a high degree of phenotypic/functional equivalency in established iPSCs-NPCs to fetal tissue-derived or ES-derived NPCs after long-term *in vivo* grafting. More recently, using SeV reprogrammed adult pig skin fibroblasts-derived iPSC-NPCs, we have demonstrated a comparable long-term (2–8 months) engraftment and functionality of NPCs after intra-striatal grafting in immunodeficient rat or after spinal grafting in syngeneic naive pig or allogeneic transiently immunosuppressed pig with previous spinal cord traumatic injury^36^.

In summary, we demonstrate a successful generation of expandable SeV-reprogrammed hiPSCs-NPCs line. Long-term expanded NPCs showed a stable karyogram and expression of typical neural precursor markers. *In vitro*–induced NPCs differentiate toward functional neurons, astrocytes, and oligodendrocytes. Long-term (6 months) *in vivo*–grafted iPSC-NPCs show a comparable engraftment/differentiation profile and acceptable safety as previously reported for fetal tissue–derived or ES-derived clinical-grade NPCs. Accordingly, this hiPSCs-NPCs cell line and/or NPCs-selection protocol may represent an effective technology for generating clinical-grade NPCs to be used in the perspective human clinical trial(s) in the treatment of several spinal neurodegenerative disorders such as spinal ischemic/traumatic injury, ALS, or multiple sclerosis.

## Supplemental Material

sj-jpg-1-cll-10.1177_09636897231163232 – Supplemental material for Derivation of Sendai-Virus-Reprogrammed Human iPSCs-Neuronal Precursors: In Vitro and In Vivo Post-grafting Safety Characterization Click here for additional data file. Supplemental material, sj-jpg-1-cll-10.1177_09636897231163232 for Derivation of Sendai-Virus-Reprogrammed Human iPSCs-Neuronal Precursors: In Vitro and In Vivo Post-grafting Safety Characterization by Michiko Shigyo, Yoshiomi Kobayashi, Oleksandr Platoshyn, Silvia Marsala, Tomohisa Kato Jr, Naoki Takamura, Kenji Yoshida, Akiyoshi Kishino, Mariana Bravo-Hernandez, Stefan Juhas, Jana Juhasova, Hana Studenovska, Vladimir Proks, Joseph D. Ciacci and Martin Marsala in Cell Transplantation

sj-jpg-2-cll-10.1177_09636897231163232 – Supplemental material for Derivation of Sendai-Virus-Reprogrammed Human iPSCs-Neuronal Precursors: In Vitro and In Vivo Post-grafting Safety Characterization Click here for additional data file. Supplemental material, sj-jpg-2-cll-10.1177_09636897231163232 for Derivation of Sendai-Virus-Reprogrammed Human iPSCs-Neuronal Precursors: In Vitro and In Vivo Post-grafting Safety Characterization by Michiko Shigyo, Yoshiomi Kobayashi, Oleksandr Platoshyn, Silvia Marsala, Tomohisa Kato Jr, Naoki Takamura, Kenji Yoshida, Akiyoshi Kishino, Mariana Bravo-Hernandez, Stefan Juhas, Jana Juhasova, Hana Studenovska, Vladimir Proks, Joseph D. Ciacci and Martin Marsala in Cell Transplantation

sj-jpg-3-cll-10.1177_09636897231163232 – Supplemental material for Derivation of Sendai-Virus-Reprogrammed Human iPSCs-Neuronal Precursors: In Vitro and In Vivo Post-grafting Safety Characterization Click here for additional data file. Supplemental material, sj-jpg-3-cll-10.1177_09636897231163232 for Derivation of Sendai-Virus-Reprogrammed Human iPSCs-Neuronal Precursors: In Vitro and In Vivo Post-grafting Safety Characterization by Michiko Shigyo, Yoshiomi Kobayashi, Oleksandr Platoshyn, Silvia Marsala, Tomohisa Kato Jr, Naoki Takamura, Kenji Yoshida, Akiyoshi Kishino, Mariana Bravo-Hernandez, Stefan Juhas, Jana Juhasova, Hana Studenovska, Vladimir Proks, Joseph D. Ciacci and Martin Marsala in Cell Transplantation

sj-jpg-4-cll-10.1177_09636897231163232 – Supplemental material for Derivation of Sendai-Virus-Reprogrammed Human iPSCs-Neuronal Precursors: In Vitro and In Vivo Post-grafting Safety Characterization Click here for additional data file. Supplemental material, sj-jpg-4-cll-10.1177_09636897231163232 for Derivation of Sendai-Virus-Reprogrammed Human iPSCs-Neuronal Precursors: In Vitro and In Vivo Post-grafting Safety Characterization by Michiko Shigyo, Yoshiomi Kobayashi, Oleksandr Platoshyn, Silvia Marsala, Tomohisa Kato Jr, Naoki Takamura, Kenji Yoshida, Akiyoshi Kishino, Mariana Bravo-Hernandez, Stefan Juhas, Jana Juhasova, Hana Studenovska, Vladimir Proks, Joseph D. Ciacci and Martin Marsala in Cell Transplantation
