# Supplementary figures and images for: Derivation of Sendai-Virus-Reprogrammed Human iPSCs-Neuronal Precursors: In Vitro and In Vivo Post-grafting Safety Characterization
Source: Cell Transplant. 2023 Mar 23;32:09636897231163232. doi: 10.1177/09636897231163232 (PMC10041596; doi:10.1177/09636897231163232)

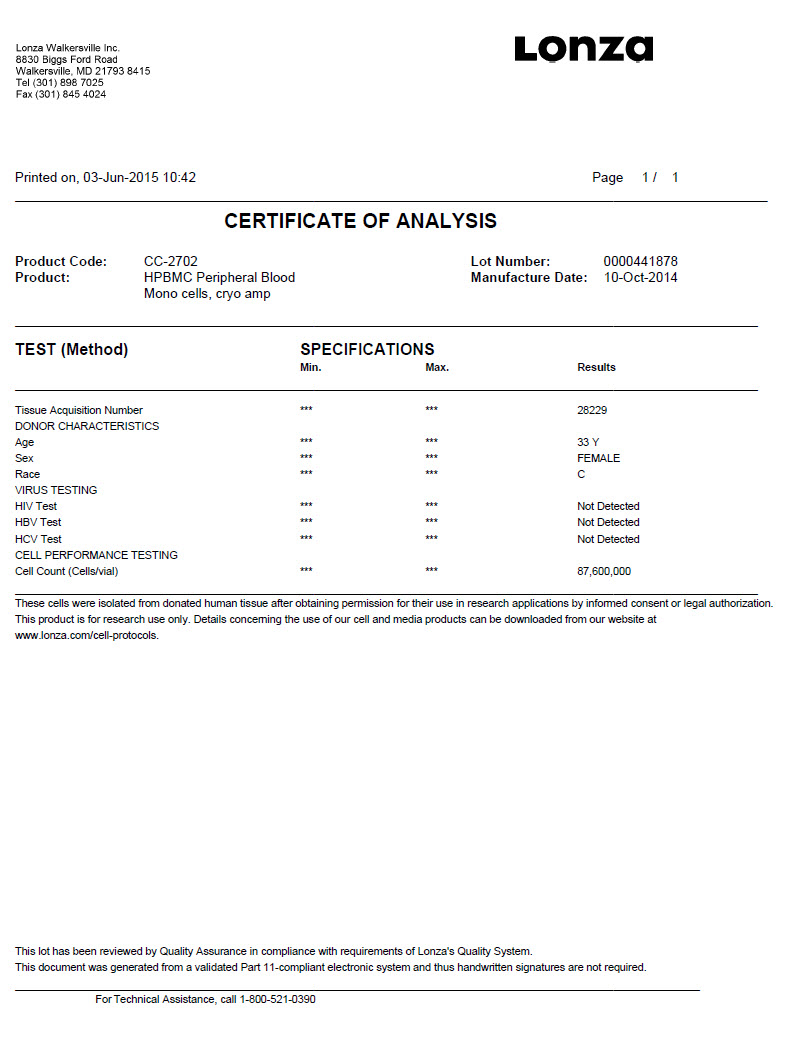

Supplement: sj-jpg-1-cll-10.1177_09636897231163232 – Supplemental material for Derivation of Sendai-Virus-Reprogrammed Human iPSCs-Neuronal Precursors: In Vitro and In Vivo Post-grafting Safety Characterization [file sj-jpg-1-cll-10.1177_09636897231163232.jpg]

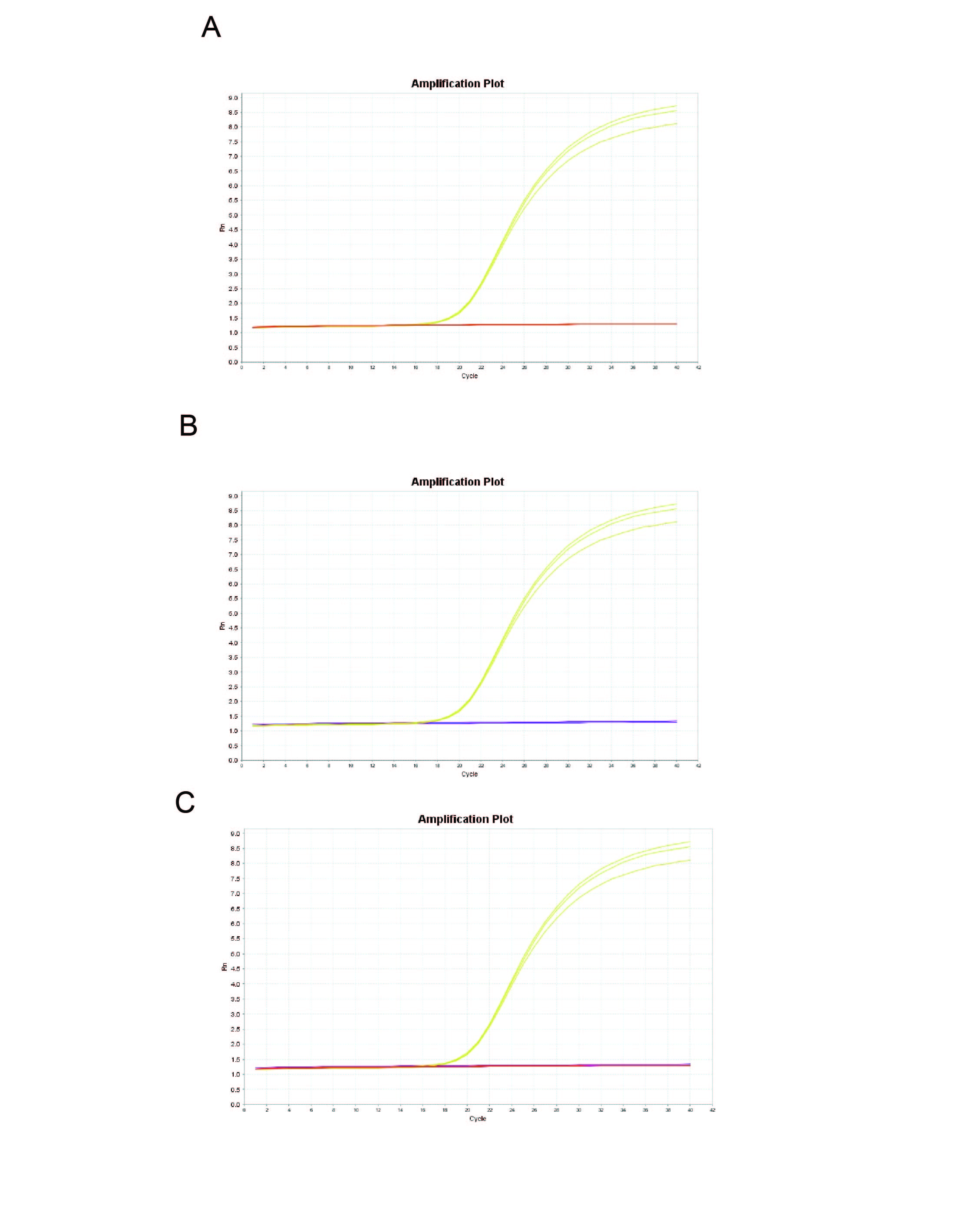

Supplement: sj-jpg-2-cll-10.1177_09636897231163232 – Supplemental material for Derivation of Sendai-Virus-Reprogrammed Human iPSCs-Neuronal Precursors: In Vitro and In Vivo Post-grafting Safety Characterization [file sj-jpg-2-cll-10.1177_09636897231163232.jpg]

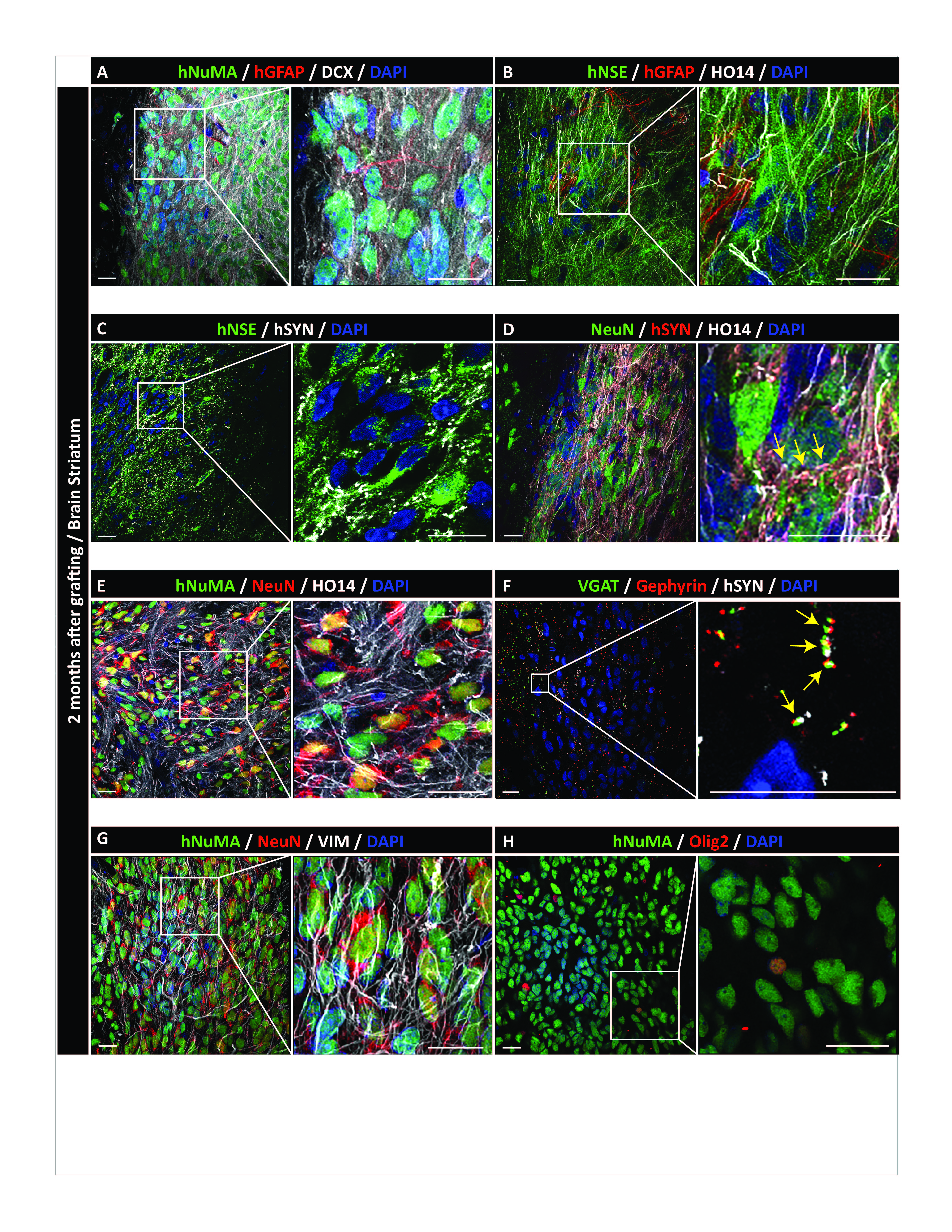

Supplement: sj-jpg-3-cll-10.1177_09636897231163232 – Supplemental material for Derivation of Sendai-Virus-Reprogrammed Human iPSCs-Neuronal Precursors: In Vitro and In Vivo Post-grafting Safety Characterization [file sj-jpg-3-cll-10.1177_09636897231163232.jpg]

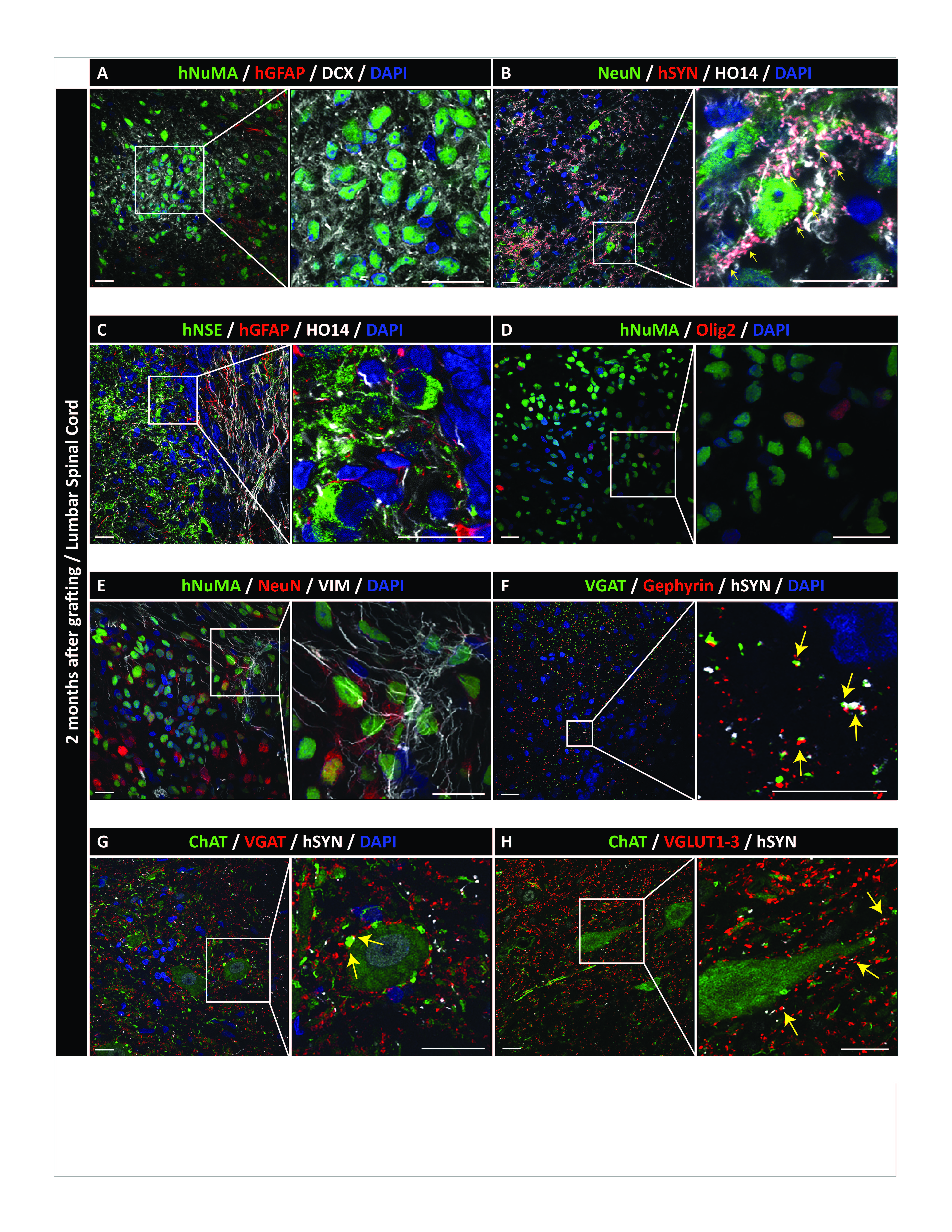

Supplement: sj-jpg-4-cll-10.1177_09636897231163232 – Supplemental material for Derivation of Sendai-Virus-Reprogrammed Human iPSCs-Neuronal Precursors: In Vitro and In Vivo Post-grafting Safety Characterization [file sj-jpg-4-cll-10.1177_09636897231163232.jpg]
